# Mycological Analysis of the Oral Cavity of Patients Using Acrylic Removable Dentures

**DOI:** 10.1155/2012/951572

**Published:** 2012-04-08

**Authors:** Bartłomiej W. Loster, Jolanta Loster, Aneta Wieczorek, Wojciech Ryniewicz

**Affiliations:** ^1^Department of Orthodontics, Institute of Dentistry at Jagiellonian University Kraków, 31-155 Kraków, Poland; ^2^Department of Dental Prosthetics, Institute of Dentistry at Jagiellonian University Kraków, 31-155 Kraków, Poland

## Abstract

*Background*. The problems of fungal infections in edentulous have been discussed in literature. Findings show that oral mycosis has an influence on the mycosis of oesophageal mucosa. Based on this we started to follow from 2007 in patients who wear dentures mycological examination, to evaluate changes of yeasts numbers, the sensitivity to antibiotics and determine the impact of types of prosthesis, time of using, gender and age of patients. 1230 patients who were wearing dentures participated in the retrospective study. The material for mycological examination was sampled as a smear from the palate. After the mycological identification of *Candida* species and assessment of growth, the susceptibility testing with Fluconazole and Nystatin was made. The number of 23 *Candida* species was diagnosed microbiologically in five years. *C. albicans* and *C. glabrata* were increasing in number—from 33,7% to 46,9% and 6,7% to 14,0%, respectively. There was a significant statistical difference between yeasts growth and gender (*P* = 0, 017 < 0.05). The conclusion is that a large percentage of persons wearing removable denture has been affected by *Candida* species and that could lead to the mycosis of farther gastrointestinal tract sections. The mycological examination before treatment, especially in patients using acrylic denture, appears to be necessary.

## 1. Introduction

*Candida *species are a normal oral commensal present in 30%–60% of healthy people. They are found in the oral cavity of 60%–100% of denture-wearing persons. *Candida albicans* is the most common species, for almost 70% of the isolates [1–3]. It is regarded as leading of oral candidiasis which is called denture stomatitis [4, 5]. Infections of *Candida* are common and often recurrent and play an important role in numerous clinical problems [6]. Finding *Helicobacter pylori* inside the yeast *Candida* could play a significant task in the bacterial reinfection of stomach and could be the reservoir of *Helicobacter pylori* in transmission to a new host. This might explain the persistence of *Helicobacter pylori* in the gastrointestinal tract [7, 8]. On the other hand, findings by Loster et al. [9] show that oral mycosis in complete dentures users has an essential influence on the mycosis of oesophageal mucosa, and the treatment of oral mycosis should be coordinated with systemic treatment of farther sections of the gastrointestinal tract, prescribed by the gastroenterologist. Our findings as well as Nikawa's ones revealed that oral mycosis in denture users has an influence on the mycosis of oesophageal mucosa [9–11].

Based of above-mentioned findings, from January 2007, we started to follow the procedure of mycological examination of patients, who wear acrylic dentures and would like to have a new removable denture [12]. The aim of our study was the mycological evaluation of the oral cavity of patients wearing partial and complete acrylic dentures. Our primary purpose was to evaluate changes of yeasts numbers during the time of five years and the sensitivity to antibiotics. We also aimed to determine the impact of types of dental prosthesis, time of using them, the gender and age of patients to different species of yeasts and the amount of growth.

## 2. Materials and Methods

A retrospective review occurred for a period just under 5 years—from January 15, 2007 to November 15, 2011. The patients wearing removable dentures in upper jaw, before dental treatment at the Department of Prosthodontics, University Dental Clinic (UDC) in Krakow were selected for this study. From this analysis we excluded patients with postoperative or framework dentures and persons with lack of necessary data. Each participant was requested to answer the questions about their age, gender, age of the prosthesis, and general health status. Evaluation of the denture was made by direct examination. Oral health status of all individuals was evaluated by the specialists of prosthetic dentistry. All data were collected in individual electronic medical history of patients using integrated software system KS-SOMED produced by KAMSOFT S.A. (Katowice, Poland).

### 2.1. Samples and Techniques

**Figure 1:**
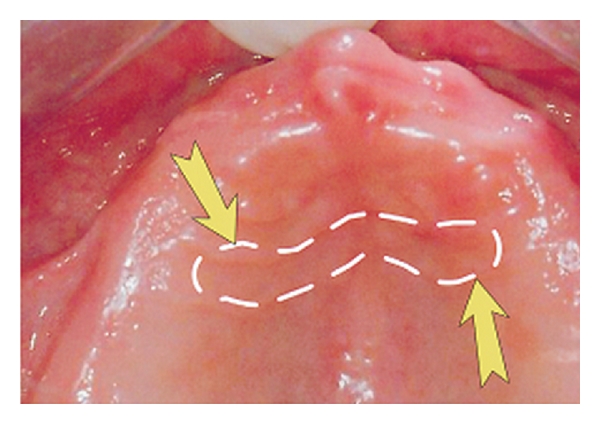
Sampling site of the material for mycological examinations. The dashed line on the photograph indicates the “sampling site” localized between the anatomic second and third palatal folds.

The material for mycological testing was collected by the uniform protocol [9, 13]. Before the examination patients were instructed to avoid any hygienic habits from the evening before. The dentures were overnight wearing. No feeding, drinking, smoking, and removing the denture before the examination which took place between 8 AM and 10 AM was required. The material was sampled as a smear from the special region of palate. Sterile cotton swab was moistened by saline and rubbed by a rotating of palate between second and third palatal folds, immediately after removal of the upper denture (Figure 1).

**Figure 2:**
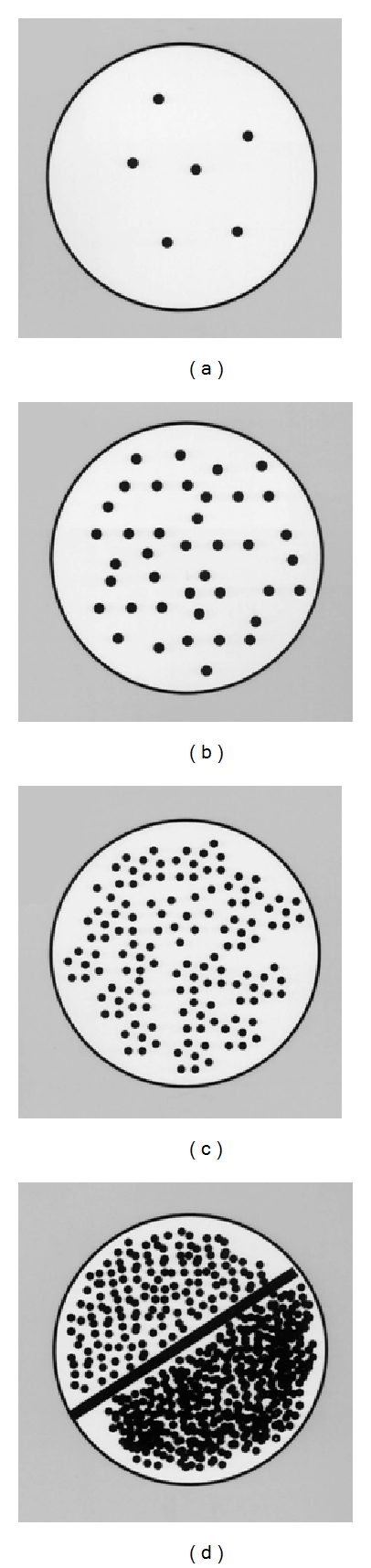
Fungal growth intensities: (a) (+)—mild growth; (b) (++)—intermediate growth; (c) (+++)—intensive growth; (d) (++++)—abundant (confluent) growth.

After this step, a swab was inserted in to the tube with Steward transport medium and sent to mycological laboratory in Microbiology Department of Krakow University Hospital. In the laboratory, the material was immediately transferred to Sabouraud medium. It was incubated at 37°C and inspected after 24 and 48 hours. The intensity of infection was classified by quantitative assessment, as follows: (0) a lack of fungal growth up to 10 colonies; (1) + scarce growth—10 to 20 colonies; (2) ++ intermediate growth—20 to 50 colonies; (3) +++ intensive growth—50 to 100 colonies; (4) ++++ abundant (confluent) growth—over 100 colonies (Figure 2).

Next step depended on the intensity of growth. Yeast colonies from 10 to 20 (+ scarce growth) were identified by colony color on the CHROMAgar medium as follows: *Candida albicans* or species non-*albicans*. In other cases (++ intermediate, +++ intensive, and ++++ abundant growth), preliminary identification of the species was made by colony color on the CHROMAgar medium, and after receiving not only *Candida albicans* species, but also commercially available validation system, the Vitek 2 Compact automated identification with YST cards (bioMerieux SA, Lyon, France) has been used from June 2009 till now. From January 2007 to May 2009 single morphotypes were identified using ID 32 C testing strips, following with manufacturer's instructions (bioMerieux SA, Lyon, France).

### 2.2. Antifungal Susceptibility Testing

 Susceptibility testing was made for cases of ++ intermediate, +++ intensive, and ++++ abundant growth, using Fungi test to evaluate the flukonazole sensitivity, and was performed according to the manufacturer's instruction (Bio-Rad Marnes-la-Coquette, France). The results were interpreted as sensitive, intermediate sensitive, or inhibited. Sensitivity to Nystatin was determined by the disk-diffusion method with Nystatin 100 units (EMAPOL Gdańsk, Poland) on the Mueller Hinton Agar, after 24 h of incubation in 37°C. The zone of inhibition of growth bigger than 18 mm was classified as drug sensitive, smaller as intermediate sensitive, and lack of inhibition of growth as inhibited.

**Figure 3:**
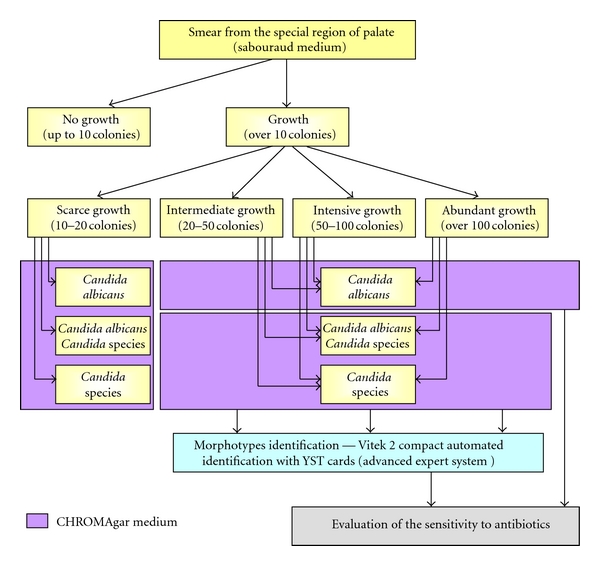
Diagram of microbiological procedure.

 Full microbiological procedure was presented on the Figure 3.

### 2.3. Statistical Analysis

 Statistical analysis was carried out using SPSS (Statistical Package for Social Sciences) for windows.

 Obtained data were evaluated by Kruskal-Wallis test, chi-squared test, Mann-Whitney *U* test, and Student's *t*-test. Crosstabulation was prepared. In all the statistical tests, a value of *P* < 0,05 was considered significant.

## 3. Results

1230 patients (832 females and 398 males), who were wearing full or partial dentures, participated in the study (Table 1).

In each year of examination the number of treated females was almost twice as big as males (Table 2).

The number of males and females in groups of age was statistically significant (*P* < 0,005). Significantly more females in each age interval were treated, especially from 41 to 60 years of age.

The patients were in age from 25 to 90 years, mean age 64. The relationship between age groups and general health status, like hypertension (*P* = 0, 000 < 0,05) and diabetes (*P* = 0, 019 < 0,05), was statistically significant, with the biggest number of these health problems between 61 to 70 years of age in both groups, respectively. Chronic peptic ulcer disease (*P* = 0, 631 > 0,05) and depression (*P* = 0, 594 > 0,05) with relation to age groups were not statistically significant.

The average age of full denture users was 65,5 and of partial denture wearers 62,6 years.

The number of full and partial denture users in groups of patients age is in Table 4. There was a statistical difference between age groups (*P* < 0,05). Significantly more patients in age 61–70 were wearing full (24,4%) and partial dentures (10,2%).

In each year of examination the number of patients with full denture was bigger than with partial denture. No relationship was determined between number of full or partial dentures users according to the year of examination (*P* = 0,843 > 0,05) (Table 5).

The time of using denture oscillated from 1 to 37 years, mean time for full denture was 8,4, and for partial denture 6,9 years. There was a statistical difference between time of denture using in Mann-Whitney *U* test (*P* = 0,000 < 0,05). Statistically longer full denture than partial one has been used.

The relationship between type of denture and general health status like hypertension (*P* = 0, 662 > 0,05), diabetes (*P* = 0, 944 > 0,05), chronic peptic ulcer disease (*P* = 0, 174 > 0,05), and depression (*P* = 0, 723 > 0,05) was not statistically significant.

Table 6 shows yeasts species isolated from palatal mucosa samples taken from the individuals using different types of prostheses in 5-years time. The number of 23 species was microbiologically diagnosed. In almost one-third of examined cases no yeasts growth was found. *C. albicans* was prime growing agent in 571 cases (41,1%). In more than 10% of the cultures, *C. glabrata* was isolated. In 5,1% of total growth, *C. species non-albicans *was found without identification of single morphotypes.

In Table 7 distribution of culture results according to the years of examination has been presented. The number of patients without yeasts varies between 30,9% and 41,3%. *C. albicans* has been found in 33,7% cases in 2008 up to 46,9% in 2010. Increasing number of *C. glabrata* has been observed—from 6,7% cases in 2007 to 14,0% in 2011. More than 1% of total amount of yeasts was *C. tropicalis* but the number of that species decreased from 3,3% in 2007 to 1,2% in 2011. Despite previously discussing number of *C. species non-albicans*, the only yeasts isolated in amount bigger than 1% of the year of examination were *C. inconspicua* in 2010 (1,4%) and *C. krusei *in 2011 (1,2%). Remaining yeasts were isolated in quantities of less than 1% per year of analysis.

The intensity of infection of yeasts growth has been found. The amount of each of them according to the years of examination has been presented in Table 8. There was a statistical difference between years of examination (*P* = 0,000 < 0,05). Significantly more intermediate, intensive and abundant growth of yeasts has been found in 2011 (52%) and increasing tendency has been observed during the years of examination.

The amount of yeasts growth according to the gender has been presented in Table 9. There was a significant statistical difference between yeasts growth and gender (*P* = 0,017 < 0,05). In females fungus, growth was bigger in scare (20%) and in intermediate, intensive, and abundant growth (45,9%).

The distribution of number of yeasts colony according to the type of denture used has been presented in Tables 10 and 11. Type of denture use was a determining factor for number of yeast colony growth. If patients were full denture wearers, one yeast colony was more often found than two or more of them (*P* = 0,006 < 0,05) even if compared with no growth (*P* = 0,022 < 0,05).

 In Table 12 the distribution of number of yeasts colony and no growth according to the year of examination has been presented. There was a significant statistical difference between years (*P* = 0,002 < 0,05).

**Figure 4:**
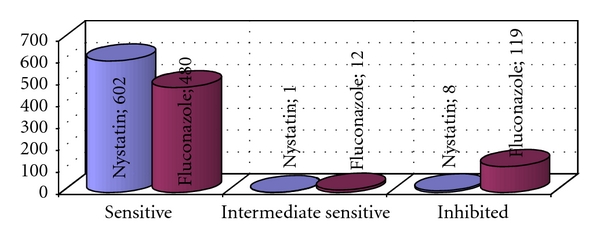
Susceptibility of yeasts of intermediate, intensive, and abundant growth to Nystatin and Flukonazole.

Susceptibility testing was made for 611 cases of intermediate, intensive, and abundant yeast growth to Fluconazole and Nystatin. Sensitivity (intensive and intermediate) to Fluconazole has been found in 80,5% of cases, to Nystatin, respectively, in 98,7% of yeast (Figure 4).

## 4. Discussion

Healthy individuals can carry *Candida* yeasts in the oral cavity without oral candidiasis. *Candida* species occur as commensal microorganisms in about 25–50% of adults with natural teeth. Their increase in number is gaining pathogenicity [1, 2, 14, 15]. In denture-wearing persons *Candida* yeasts are found in the oral cavity of 60%–100% of them. Many denture-using patients are unaware that they have oral candidosis. Because of this, from January 2007, we started to follow mycological examination of all patients, who wear acrylic dentures and would like to have a new removable denture in our Prosthodontic Department.

After five years of examination we carried out findings in 1230 patients. They were using 852 full or 378 partial dentures in upper jaw. Some of them were using lower dentures as well, but it was not the subject of this study. In each year we have investigated a similar number of patients. But the number of females was twice bigger than males in every year. In each analyzed age group the number of women was bigger (with statistical significance) (Table 3). Probably it was because of aesthetic needs.

In all five years, the number of full denture-wearing patients was almost twice bigger than partial denture users. The biggest number of full and partial denture-wearing individuals were in age between 51 and 80, but peak volume was from 61 to 70 years of age (Table 4). In that years of age, in our study, significantly more patients complained of hypertension and diabetes. This may mean that they use medicines which could decrease amount of saliva (in hypertension) or have angiopathy (in diabetes) and theirs mucous membrane could be not properly feeding [2]. Mandali et al. [16] reported that age was one of the determining factors of denture stomatitis. Hand and Whitehill noted that the elderly was the only significant factor in the risk of denture-associated lesions [17]. Similar observation reported by Jainkittivong et al. [18] was that the incidence of oral mucosal conditions indicated a significant increase with advancing age. Kossioni reported the opposite result in Greeks population [19]. She did not observe a significant relation between the age of participants and denture-related stomatitis.

In this study time of using full denture was significantly longer than partial ones. The longest time of wearing full denture was 37 years, but the mean time of use of the same denture was 7,9 years. In Lyon et al. study, the mean time of wearing the same denture was 10,5 years [2]. Time of denture using has been considered as associated with denture stomatitis [16, 20, 21]. But Abaci et al. [14] did not find a significant relationship between denture stomatitis and increasing age of the denture.

Our results revealed the presence of 23 *Candida* species in five years of examination of acrylic denture users palatal mucosa. *Candida albicans* was the most frequently recognized, in 79,31% of all the cases with overgrowth of yeast. Other common palatal *Candida* species non-*albicans* were *Candida glabrata* (20,83%) and *Candida tropicalis* (4,44%). *Candida *species non-*albicans* which has been diagnosed in cases with scarce growth, without morphotype identification, accounted 9,87% of all yeasts culture. In total amount of patients yeasts, *Candida albicans* and *Candida *species non-*albicans* have been found in 58,54% of investigation. Other yeasts have been identified in very few, smaller than 1% (Tables 6 and 7) in amount of year of examination.

It is not unequivocal to compare our findings with other studies, because of methods of taking samples. Some authors inspected oral rinse samples or unstimulated saliva [5, 14], mucosal side of denture or denture sonication [1, 5, 22–24], samples of the dorsum of the tongue and/or palatal mucosa [2, 23]. We know that *Candida* species, and especially *Candida albicans,* has an adhesive capacity and generates biofilms, but for patients' oral health the mucosal status is the most important. It has been shown that adhesion of the mycelium form of *Candida albicans* to epithelial cells is stronger, proteinases secreted by that form cause damage in oral mucosa [15]. The denture could be taken out or relining, or changing. Because of that, sampling of swabs of palatal mucosa appears to be the best way of examination.

In our study we did not exclude patients with general health problems even with denture stomatitis, like same other authors [1, 2, 14]. Patients were examined irrespective of oral mucosal status. As many authors reported, candidiasis occurs regardless of clinical signs [1, 9, 19, 20, 23].

The intensity of yeast infection was classified by quantitative assessment and compared with the year of examination and gender. We observed significantly more intermediate, intensive, and abundant growth of yeasts in 2011 (52%) and increasing tendency has been found during the years of examination (Table 8). The results of study of Salmanian et al. [7] propose that oral yeast could provide the shelter for *Helicobacter pylori. Candida* species are well known as highly sophisticated microorganisms that possess fascinating plasticity for rapid adaptation to environmental challenges. It is concluded that yeast, which is found in oral cavity, as well as gastrointestinal tract of human, could be the reservoir of *Helicobacter pylori* and play an important role in the bacterial reinoculation of farther gastrointestinal tract sections. Based on this information, it is important to diagnose patients who are users of removable denture, even asymptomatic, due to the importance of the oral mucosa as a primary source of reinfection.

We found significant correlation between gender. In females yeasts growth was bigger in amount of intensity of growth (*P* = 0,017). Lyon et al. [2] reported that factors like gender, advanced age, and xerostomia were associated with presence of yeasts. Webb et al. [23] found in his studies conducted in this topic that development of denture-related stomatitis in women was higher due to hormonal changes. Abaci et al. [14] did not find statistical difference in gender related to the amount of yeast and denture stomatitis.

As we presented in Tables 6 and 7 not only *Candida albicans* has been found in our study. The correlation of number of yeast colony with type of wearing denture has been shown in Tables 10 and 11. Patients wearing full denture more often have one colony of *Candida* species, and in 5 years evaluation one colony of yeast has been found more often (excluding year 2008 when no yeast growth has been identified in biggest number of cases); it can be noted that the number of two and more *Candida's* colony is increasing (Table 12). The occurrence of more than one *Candida species* colony has been reported by many authors [1, 2, 4, 5, 9, 14, 15, 20, 22, 23, 25].

Susceptibility testing to Fluconazole and Nystatin has found very high sensitivity of 611 cases (Figure 4). Due to the specifics of pharmaceutical market in Poland, Nystatin is easily available for patients, our results indicate the possibility of an effective treatment. The effectiveness of antifungal therapy in the treatment of denture stomatitis with Nystatin has been reported by Bergendal and Isaccson [24], DePaola et al. [26], and also Gendreau and Loewy [20]. Due to the opposite results of *in vitro* findings by Chandra et al. [27], further investigations should be carried out.

## 5. Conclusion

A large percentage of persons wearing removable denture has been affected by *Candida* species and that could lead to the mycosis of farther gastrointestinal tract sections. The mycological examination before treatment, especially in patients using acrylic denture, appears to be necessary.

## Figures and Tables

**Table 1:** Distributions of dentures according to gender.

		Gender		Total
		Male	Female	
Full denture	Amount	279	573	852
	% from total	22,7%	46,6%	69,3%
Partial denture	Amount	119	259	378
	% from total	9,7%	21,1%	30,7%

Total	Amount	398	832	1230
	% from total	32,4%	67,6%	100,0%

**Table 2:** Distributions of gender according to the year of examination.

		Year of examination					Total
		2007	2008	2009	2010	2011	
Male	Amount	81	93	66	65	93	398
	% from total	6,6%	7,6%	5,4%	5,3%	7,6%	32,4%

Female	Amount	147	177	149	178	181	832
	% from total	12,0%	14,4%	12,1%	14,5%	14,7%	67,6%

Total	Amount	228	270	215	243	274	1230
	% from total	18,5%	22,0%	17,5%	19,8%	22,3%	100,0%

**Table 3:** Distributions of gender according to the age groups.

		Age groups						Total
		<40 years	41–50 years	51–60 years	61–70 years	71–80 years	>80 years	
Male	Amount (% of age group)	6 (32%)	21 (24%)	79 (26%)	142 (34%)	124 (39%)	26 (40%)	398
Female	Amount (% of age group)	14 (68%)	67 (76%)	235 (74%)	284 (66%)	193 (61%)	39 (60%)	832

Total amount		20	88	314	426	317	65	1230

Chi-squared Pearson's: 0,003.

**Table 4:** The numbers of denture users in groups of age.

		Age groups						Total
		<40 years	41–50 years	51–60 years	61–70 years	71–80 years	>80 years	
Full denture	Amount	7	54	204	300	233	54	852
	% from total	0,6%	4,4%	16,6%	24,4%	18,9%	4,4%	69,3%

Partial denture	Amount	13	34	110	126	84	11	378
	% from total	1,1%	2,8%	8,9%	10,2%	6,8%	0,9%	30,7%

Total	Amount	20	88	314	426	317	65	1230
	% from total	1,6%	7,2%	25,5%	34,6%	25,8%	5,3%	100,0%

Chi-squared Pearson's: 0,000.

**Table 5:** Distributions of dentures according to the year of examination.

		Year of examination					Total
		2007	2008	2009	2010	2011	
Full denture	Amount	158	189	152	161	192	852
	% from total	12,8%	15,4%	12,4%	13,1%	15,6%	69,3%

Partial denture	Amount	70	81	63	82	82	378
	% from total	5,7%	6,6%	5,1%	6,7%	6,7%	30,7%

Total	Amount	228	270	215	243	274	1230
	% from total	18,5%	22,0%	17,5%	19,8%	22,3%	100,0%

**Table 6:** Yeasts species isolated from palatal mucosa.

		Frequencies	Percent
(1)	No yeasts	510	36,7
(2)	*C. albicans*	571	41,1
(3)	*C. glabrata*	150	10,8
(4)	*C. tropicalis*	32	2,3
(5)	*C. *species non*-albicans *	71	5,1
(6)	*C. parapsilosis*	5	0,4
(7)	*C. rugosa*	4	0,3
(8)	*C. lusitaniae*	3	0,2
(9)	*C. dubliniensis*	6	0,4
(10)	*C. intermedia*	2	0,1
(11)	*C. inconspicua*	8	0,6
(12)	*Saccharomyces cerevisiae*	3	0,2
(13)	*C. pseudotropicalis*	1	0,1
(14)	*C. kefyr*	4	0,3
(15)	*C. ciferrii*	1	0,1
(16)	*C. guilliermondii*	1	0,1
(17)	*C. krusei*	8	0,6
(18)	*C. famata*	2	0,1
(19)	*Geotrichum *species	1	0,1
(20)	*Saccharomyces kluyveri*	1	0,1
(21)	*C. lipolytica*	2	0,1
(22)	*C. lambica*	1	0,1
(23)	*C. norvegensis*	1	0,1
(24)	*C. boidinii *	1	0,1
(25)	*Rhodotorula glutinis*	1	0,1

	Total grow	1390	100,0

**Table 7:** Distribution of yeasts results according to the year of examination.

		Year of examination					Total
		2007	2008	2009	2010	2011	
No yeasts	Amount	99	125	84	89	113	510
	% of year of exam.	41,3%	41,3%	35,3%	30,9%	35,2%	36,7%
*C. albicans*	Amount	98	102	98	135	138	571
	% of year of exam.	40,8%	33,7%	41,2%	46,9%	43,0%	41,1%
*C. glabrata*	Amount	16	34	26	29	45	150
	% of year of exam.	6,7%	11,2%	10,9%	10,1%	14,0%	10,8%
*C. tropicalis*	Amount	8	6	9	5	4	32
	% of year of exam.	3,3%	2,0%	3,8%	1,7%	1,2%	2,3%
*C. *species non*-albicans *	Amount	10	25	15	17	4	71
	% of year of exam.	4,2%	8,3%	6,3%	5,9%	1,2%	5,1%
*C. parapsilosis*	Amount	2	0	0	1	2	5
	% of year of exam.	0,8%	0,0%	0,0%	0,3%	0,6%	0,4%
*C. rugosa*	Amount	0	1	0	2	1	4
	% of year of exam.	0,0%	0,3%	0,0%	0,7%	0,3%	0,3%
*C. lusitaniae*	Amount	1	1	1	0	0	3
	% of year of exam.	0,4%	0,3%	0,4%	0,0%	0,0%	0,2%
*C. dubliniensis*	Amount	0	1	2	1	2	6
	% of year of exam.	0,0%	0,3%	0,8%	0,3%	0,6%	0,4%
*C. intermedia*	Amount	0	1	0	0	1	2
	% of year of exam.	0,0%	0,3%	0,0%	0,0%	0,3%	0,1%
*C. inconspicua*	Amount	0	2	0	4	2	8
	% of year of exam.	0,0%	0,7%	0,0%	1,4%	0,6%	0,6%
*Saccharomyces cerevisiae*	Amount	0	1	0	1	1	3
	% of year of exam.	0,0%	0,3%	0,0%	0,3%	0,3%	0,2%
*C. pseudotropicalis*	Amount	1	0	0	0	0	1
	% of year of exam.	0,4%	0,0%	0,0%	0,0%	0,0%	0,1%
*C. kefyr*	Amount	0	1	2	0	1	4
	% of year of exam.	0,0%	0,3%	0,8%	0,0%	0,3%	0,3%
*C. ciferrii*	Amount	0	0	1	0	0	1
	% of year of exam.	0,0%	0,0%	0,4%	0,0%	0,0%	0,1%
*C. guilliermondii*	Amount	0	0	0	0	1	1
	% of year of exam.	0,0%	0,0%	0,0%	0,0%	0,3%	0,1%
*C. krusei*	Amount	2	0	0	2	4	8
	% of year of exam.	0,8%	0,0%	0,0%	0,7%	1,2%	0,6%
*C. famata*	Amount	0	1	0	0	1	2
	% of year of exam.	0,0%	0,3%	0,0%	0,0%	0,3%	0,1%
*Geotrichum *species	Amount	0	1	0	0	0	1
	% of year of exam.	0,0%	0,3%	0,0%	0,0%	0,0%	0,1%
*Saccharomyces kluyveri*	Amount	0	1	0	0	0	1
	% of year of exam.	0,0%	0,3%	0,0%	0,0%	0,0%	0,1%
*C. lipolytica*	Amount	1	0	0	1	0	2
	% of year of exam.	0,4%	0,0%	0,0%	0,3%	0,0%	0,1%
*C. lambica*	Amount	0	0	0	1	0	1
	% of year of exam.	0,0%	0,0%	0,0%	0,3%	0,0%	0,1%
*C. norvegensis*	Amount	0	0	0	0	1	1
	% of year of exam.	0,0%	0,0%	0,0%	0,0%	0,3%	0,1%
*C. boidinii*	Amount	1	0	0	0	0	1
	% of year of exam.	0,4%	0,0%	0,0%	0,0%	0,0%	0,1%
*Rhodotorula glutinis*	Amount	1	0	0	0	0	1
	% of year of exam.	0,4%	0,0%	0,0%	0,0%	0,0%	0,1%

Total	Amount	240	303	238	288	321	1390
	% of year of exam.	100,0%	100,0%	100,0%	100,0%	100,0%	100,0%

**Table 8:** Distribution of yeasts growth according to the year of examination.

		Year of examination					Total
		2007	2008	2009	2010	2011	
No yeasts growth	Amount % of year of exam.	99 41,3%	125 41,3%	84 35,3%	89 30,9%	113 35,2%	510 36,7%
Scarce growth (+)	Amount % of year of exam.	56 23,3%	65 21,5%	45 18,9%	62 21,5%	41 12,8%	269 19,4%
Intermediate, intensive, and abundant of growth (++, +++, ++++)	Amount % of year of exam.	85 35,4%	113 37,3%	109 45,8%	137 47,6%	167 52,0%	611 44,0%

Total	Amount % of year of exam.	240 100,0%	303 100,0%	238 100,0%	288 100,0%	321 100,0%	1390 100,0%

Chi-squared Pearson's: 0,000.

**Table 9:** Distribution of yeasts growth according to gender.

		Gender		Total
		Male	Female	
No yeasts growth	Amount % of gender	189 42,0%	321 34,1%	510 36,7%
Scarce growth (+)	Amount % of gender	81 18,0%	188 20,0%	269 19,4%
Intermediate, intensive, and abundant of growth (++, +++, ++++)	Amount % of gender	180 40,0%	431 45,9%	611 44,0%

Total	Amount % of gender	450 100,0%	940 100,0%	1390 100,0%

Chi-squared Pearson's: 0,017.

**Table 10:** Distribution of number of yeasts colony according to the type of denture.

		Full denture	Partial denture	Total
1 yeast colony	Amount	379	188	567
	% from total	52,6%	26,1%	78,8%
2 and more colonies	Amount	120	33	153
	% from total	16,7%	4,6%	21,3%

Total	Amount	499	221	720
	% from total	69,3%	30,7%	100,0%

Chi-squared Pearson's: 0,006.

**Table 11:** Distribution of number of yeasts colony and no growth according to the type of denture.

		Full denture	Partial denture	Total
No yeast colony	Amount % from total	353 28,7%	157 12,8%	510 41,5%
1 yeast colony	Amount % from total	379 30,8%	188 15,3%	567 46,1%
2 and more colonies	Amount % from total	120 9,8%	33 2,7%	153 12,4%

Total	Amount % from total	852 69,3%	378 30,7%	1230 100,0%

Chi-squared Pearson's: 0,022.

**Table 12:** Distribution of number of yeasts colony and no growth according to the year of examination.

		Year of examination					Total
		2007	2008	2009	2010	2011	
No yeast colony	Amount	100	125	84	89	112	510
	% of year of exam.	43,9%	46,3%	39,1%	36,6%	40,9%	41,5%
1 yeast colony	Amount	116	113	108	112	118	567
	% of year of exam.	50,9%	41,9%	50,2%	46,1%	43,1%	46,1%
2 and more colonies	Amount	12	32	23	42	44	153
	% of year of exam.	5,3%	11,9%	10,7%	17,3%	16,1%	12,4%

Total	Amount	228	270	215	243	274	1230
	% of year of exam.	100,0%	100,0%	100,0%	100,0%	100,0%	100,0%

Chi-squared Pearson's: 0,002.
